# Inhibition of the Na^+^/K^+^-ATPase by cardiac glycosides suppresses expression of the IDO1 immune checkpoint in cancer cells by reducing STAT1 activation

**DOI:** 10.1016/j.jbc.2022.101707

**Published:** 2022-02-09

**Authors:** Mia A. Shandell, Alina L. Capatina, Samantha M. Lawrence, William J. Brackenbury, Dimitris Lagos

**Affiliations:** 1 Department of Biology, University of York, York, United Kingdom; 2 Hull York Medical School, University of York, York, United Kingdom; 3 York Biomedical Research Institute, University of York, York, United Kingdom

**Keywords:** immune checkpoints, tumor ionic microenvironment, ouabain, digoxin, IDO1, sodium transport, ATP1A1, IDO1, indoleamine-pyrrole 2′,3′-dioxygenase 1, JAK/STAT, Janus kinase/signal transducer and activator of transcription, KYNU, kynureninase, NKA, Na^+^/K^+^-ATPase, NTC, nontargeting control, SBFI, sodium-binding benzofuran isophthalate, VGSC, voltage-gated sodium channel

## Abstract

Despite extensive basic and clinical research on immune checkpoint regulatory pathways, little is known about the effects of the ionic tumor microenvironment on immune checkpoint expression and function. Here we describe a mechanistic link between Na^+^/K^+^-ATPase (NKA) inhibition and activity of the immune checkpoint protein indoleamine-pyrrole 2′,3′-dioxygenase 1 (IDO1). We found that IDO1 was necessary and sufficient for production of kynurenine, a downstream tryptophan metabolite, in cancer cells. We developed a spectrophotometric assay to screen a library of 31 model ion transport-targeting compounds for potential effects on IDO1 function in A549 lung and MDA-MB-231 breast cancer cells. This revealed that the cardiac glycosides ouabain and digoxin inhibited kynurenine production at concentrations that did not affect cell survival. NKA inhibition by ouabain and digoxin resulted in increased intracellular Na^+^ levels and downregulation of IDO1 mRNA and protein levels, which was consistent with the reduction in kynurenine levels. Knockdown of ATP1A1, the ɑ1 subunit of the NKA and target of cardiac glycosides, increased Na^+^ levels to a lesser extent than cardiac glycoside treatment and did not affect IDO1 expression. However, ATP1A1 knockdown significantly enhanced the effect of cardiac glycosides on IDO1 expression and kynurenine production. Mechanistically, we show that cardiac glycoside treatment resulted in curtailing the length of phosphorylation-mediated stabilization of STAT1, a transcriptional regulator of IDO1 expression, an effect enhanced by ATP1A1 knockdown. Our findings reveal cross talk between ionic modulation *via* cardiac glycosides and immune checkpoint protein expression in cancer cells with broad mechanistic and clinical implications.

Understanding and treating cancer are a fundamental struggle of modern medicine. Breast cancer alone is responsible for 30% of newly diagnosed cancers in women in the United States over the last year, with more than 240,000 new cases registered only in 2019 (1). A key feature of highly metastatic breast tumors is represented by ionic imbalances characterized primarily by elevated intracellular Na^+^ and a slightly depolarized cell membrane (2). Therefore, growing attention has focused on the role of ion transport in cancer progression (3, 4).

Changes in ion transport drive a number of cellular phenotypes associated with cancer (5). Signal transduction, cytoskeletal remodeling, and cell motility underpinning cell migration (6, 7), growth and cell cycle progression (8, 9, 10), and gene expression (11, 12) can all be impacted by altered ion flux. Ion transport also defines the extracellular environment, for instance, by pH regulation (13, 14). There are higher concentrations of K^+^ and Na^+^ in the tumor microenvironment, accompanied by hypoxia and decreased pH relative to healthy tissue (3, 15, 16). Increased expression of a range of ion channels is associated with metastasis (4, 17, 18, 19).

The tumor microenvironment is generally immunosuppressive to prevent an attack by the host immune system. One way immunosuppression is achieved is through immune checkpoint proteins such as PD-L1 and IDO1 (20, 21). IDO1 catalyzes the rate-limiting step of tryptophan catabolism, mediating its conversion to *N*-formyl-kynurenine, which is then converted to downstream kynurenine metabolites. IDO1 metabolism depletes the tumor microenvironment of tryptophan, leading to T cell starvation and impaired activation (22, 23). Kynurenine metabolites induce differentiation into tolerogenic regulatory T cells through the aryl hydrocarbon receptor (24, 25, 26). Proinflammatory cytokine stimulation with TNF/IFN-γ induces IDO1 expression as a negative feedback mechanism to suppress inflammation (27, 28, 29). IFN-γ stimulation induces IDO1 expression, with STAT1, a downstream IFN-γ signaling mediator, being one of the key transcription factors involved in this process (29, 30, 31). Effects of the ionic tumor microenvironment on cancer immunity are less understood. The ionic content of tumors can itself act as an immune inhibitor by regulating T cell activation and stemness (16, 32).

Our understanding of how ionic transport affects tumor immune escape remains poorly explored. Here, we investigated how pharmacological modulation of multiple ionic transport pathways affects immune gene expression of cancer cells *in vitro*. We curated a library of 31 model ionic transport modulating compounds and assessed their activity in screens using lung and breast cancer cell lines. We selected levels of secreted kynurenine as our readout as this is directly connected with activity of IDO1, which, in addition to being an immune checkpoint protein, is also a surrogate marker of tumor cell immune activation. We found that in breast and lung cancer cell lines, cardiac glycosides decrease IDO1 expression by suppressing STAT1 activation.

## Results

### Cardiac glycosides inhibit kynurenine production in lung and breast cancer cells

**Figure 1:**
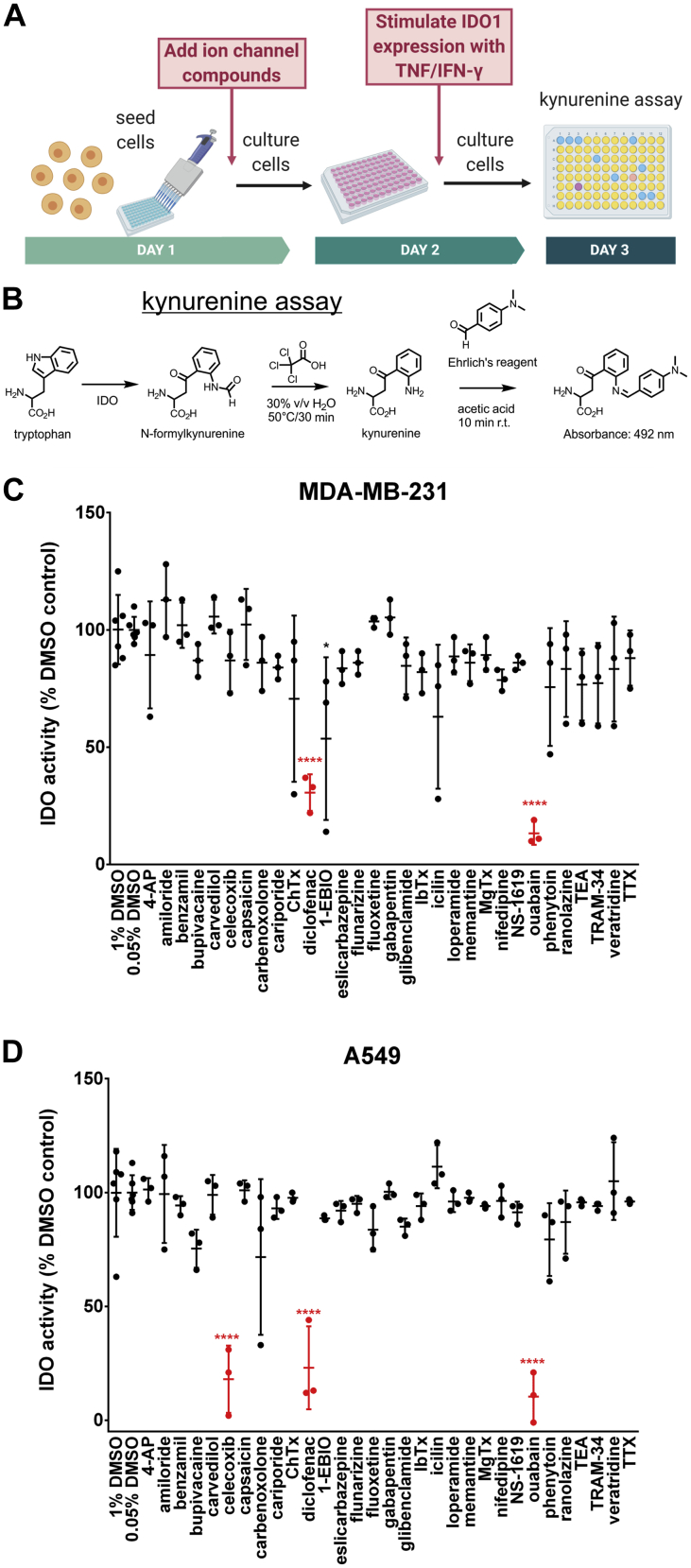
**Ion channel targeting small-molecule screen of kynurenine production.***A*, experimental setup of the ion channel targeting small molecule screen. Cell lines were treated with ion channel targeting small molecules for 24 h, then stimulated with TNF/IFN-γ for 24 h to induce IDO1 expression before assaying kynurenine production. *B*, kynurenine assay scheme. *C*, MDA-MB-231 screen results. Data were normalized to their respective DMSO control, 0.05% or 1% DMSO. ∗∗∗∗*p* < 0.0001, one-way ANOVA Tukey’s post-hoc. *D*, A549 screen results. Data were normalized to their respective DMSO control, 0.05% or 1% DMSO. ∗∗∗∗*p* < 0.0001, one-way ANOVA Tukey’s post-hoc. IDO1, indoleamine-pyrrole 2′,3′-dioxygenase 1.

To assess effects of changing the ionic environment on activity of immune checkpoint protein IDO1, we designed a screen of ion transport modulating small molecules based on colorimetric detection of kynurenines, downstream tryptophan metabolites (30) (Fig. 1, *A* and *B*). TNF/IFN-ɣ has been used to stimulate kynurenine production in human cell lines (30). Cells were pretreated with ion channel targeting small molecules for 24 h, then stimulated with TNF/IFN-ɣ for 24 h to induce IDO1 expression and kynurenine production. After drug pretreatment and cytokine stimulation, kynurenine production was measured using the colorimetric kynurenine assay (Fig. 1, *A* and *B*). We tested a library of 31 ion channel targeting compounds for their effect on kynurenine production by TNF/IFN-ɣ stimulated human breast cancer MDA-MB-231 and lung cancer A549 cells (Table S1). An initial screen of MDA-MB-231 cells resulted in decreased kynurenine (mean ± SD) upon treatment with 50 μM ouabain: 3.8 ± 1.3 μM, n = 3, *versus* 1% DMSO control: 27.5 ± 4.1 μM, n = 6 (*p* < 0.0001, one-way ANOVA Tukey’s post-hoc) (Fig. 1*C*). An initial screen of A549 cells resulted in decreased kynurenine upon treatment with 50 μM ouabain: 1.5 ± 1.6 μM (conservatively, < 3.1 μM), n = 3, *versus* 1% DMSO control: 15.0 ± 2.9 μM, n = 6 (*p* < 0.001, one-way ANOVA Tukey’s post-hoc) (Fig. 1*D*). Other hits were the cyclooxygenase-2 (COX-2) inhibitors diclofenac and celecoxib, which have been reported to reduce IDO1 expression (33, 34).

### Cardiac glycosides increase intracellular Na^+^ and inhibit IDO1 expression

**Figure 2:**
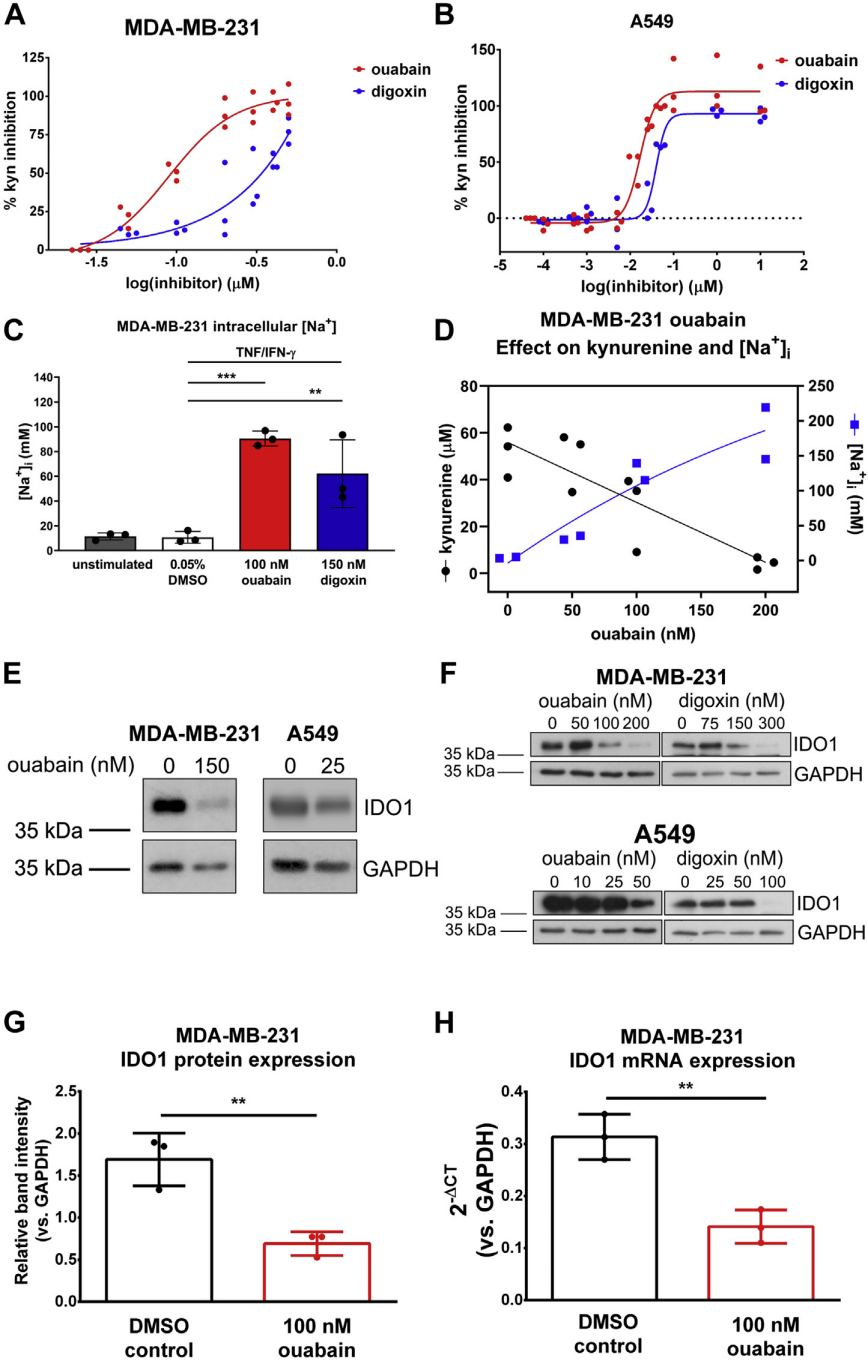
**Cardiac glycosides ouabain and digoxin inhibit kynurenine production and IDO1 protein expression in MDA-MB-231 and A549 cells.***A*, dose-response curve for ouabain and digoxin treatment of TNF/IFN-ɣ stimulated MDA-MB-231 cells. IC_50_ ouabain = 89 nM, 95% confidence interval 70 to 112 nM. Apparent IC_50_ digoxin ∼164 nM, n = 3. *B*, dose–response curve for ouabain and digoxin treatment of TNF/IFN-ɣ stimulated A549 cells. IC_50_ ouabain = 17 nM, 95% confidence interval 14 to 20 nM. IC_50_ digoxin 40 nM, 95% confidence interval 35 to 46 nM, n = 3. *C*, representative SBFI data from three independent experiments measuring intracellular sodium in response to 24-h cardiac glycoside or 0.05% DMSO control treatment of TNF/IFN-ɣ-stimulated MDA-MB-231 cells. ∗∗∗*p* < 0.001, ∗∗*p* < 0.01, One-way ANOVA, Tukey’s multiple comparisons. *D*, ouabain titration inhibits kynurenine production (3 independent experiments, •) and increases intracellular sodium ([Na^+^]_i_ (2 independent experiments, ▪) in TNF/IFN-ɣ stimulated MDA-MB-231 cells treated with the indicated concentrations of ouabain. Data were fitted using nonlinear regression by least squares fit. Pearson Correlation r^2^ = 0.9628, *p* < 0.05. *E*, representative Western blots of IDO1 protein expression in TNF/IFN-ɣ-stimulated MDA-MB-231 and A549 cells pre-treated with ouabain or 0.05% DMSO. *F*, IDO1 and GAPDH protein expression in MDA-MB-231 (*top*) or A549 (*bottom*) cells pretreated with ouabain, digoxin, or 0.05% DMSO control for 36 h, then TNF/IFN-ɣ stimulated for 24 h. Ouabain and digoxin treated samples were visualized on two different blots. *G*, relative IDO1 band intensities determined from Western blots of three independent experiments in which TNF/IFN-ɣ-stimulated MDA-MB-231 cells were pretreated with 0.05% DMSO or 100 nM ouabain. *H*, IDO1 mRNA levels from three independent experiments in which TNF/IFN-ɣ stimulated MDA-MB-231 cells were pretreated with 0.05% DMSO or 100 nM ouabain. IDO1, indoleamine-pyrrole 2′,3′-dioxygenase 1; SBFI, sodium-binding benzofuran isophthalate.

Next, we validated the effect of ouabain and the related cardiac glycoside digoxin on kynurenine production in TNF/IFN-ɣ-stimulated cancer cell lines. Titration of ouabain in the kynurenine assay resulted in an IC_50_ of 89 nM (95% confidence interval 70–112 nM) for MDA-MB-231 cells (Fig. 2*A*). Titration of digoxin resulted in an apparent IC_50_ of ∼164 nM for MDA-MB-231 cells (Fig. 2*A*). Titration of ouabain and digoxin resulted in IC_50_s of 17 nM (95% confidence interval 14–20 nM) and 40 nM (95% confidence interval 35–46 nM), respectively, for A549 cells (Fig. 2*B*). In general, ouabain was more potent than digoxin, and A549 cells were more sensitive to cardiac glycosides than MDA-MB-231 cells. Ouabain and digoxin functionally inhibit the Na^+^/K^+^ ATPase (NKA). Using a sodium-binding benzofuran isophthalate (SBFI)-based colorimetric assay, we observed increased intracellular Na^+^ in TNF/IFN-ɣ-stimulated MDA-MB-231 cells treated with cardiac glycoside concentrations near the IC_50_s measured in the kynurenine assay (100 nM ouabain or 150 nM digoxin) for 24 h (Fig. 2*C*), confirming that the two compounds inhibited Na^+^ export through targeting of the NKA in these cells. The increase in intracellular Na^+^ inversely correlated with decreasing kynurenine resulting from titration of ouabain in TNF/IFN-ɣ-stimulated MDA-MB-231 cells (Fig. 2*D*).

Having observed a reduction in kynurenine levels upon treatment with cardiac glycosides, we explored the effect of ouabain and digoxin on IDO1 protein expression and mRNA levels in TNF/IFN-ɣ-stimulated MDA-MB-231 and A549 cell lines. Cells were pretreated with concentrations of ouabain near the IC_50_ measured for each cell line. Ouabain treatment decreased IDO1 protein level in TNF/IFN-ɣ-stimulated MDA-MB-231 and A549 cells between 100 and 200 nM ouabain and between 25 and 50 nM ouabain, respectively (Fig. 2*E*). Cells were pretreated with one of three cardiac glycoside concentrations—sub-IC_50_, IC_50_, and complete inhibition—to measure the effect on IDO1 expression. Increasing concentrations of ouabain and digoxin markedly decreased IDO1 protein expression in cytokine-stimulated MDA-MB-231 and A549 cells (Fig. 2*F*). In cytokine-stimulated MDA-MB-231 cells, 100 nM ouabain treatment significantly inhibited IDO1 protein (Fig. 2*G*) and mRNA expression (Fig. 2*H*).

### IDO1 expression is necessary and sufficient for kynurenine production in MDA-MB-231 cells

**Figure 3:**
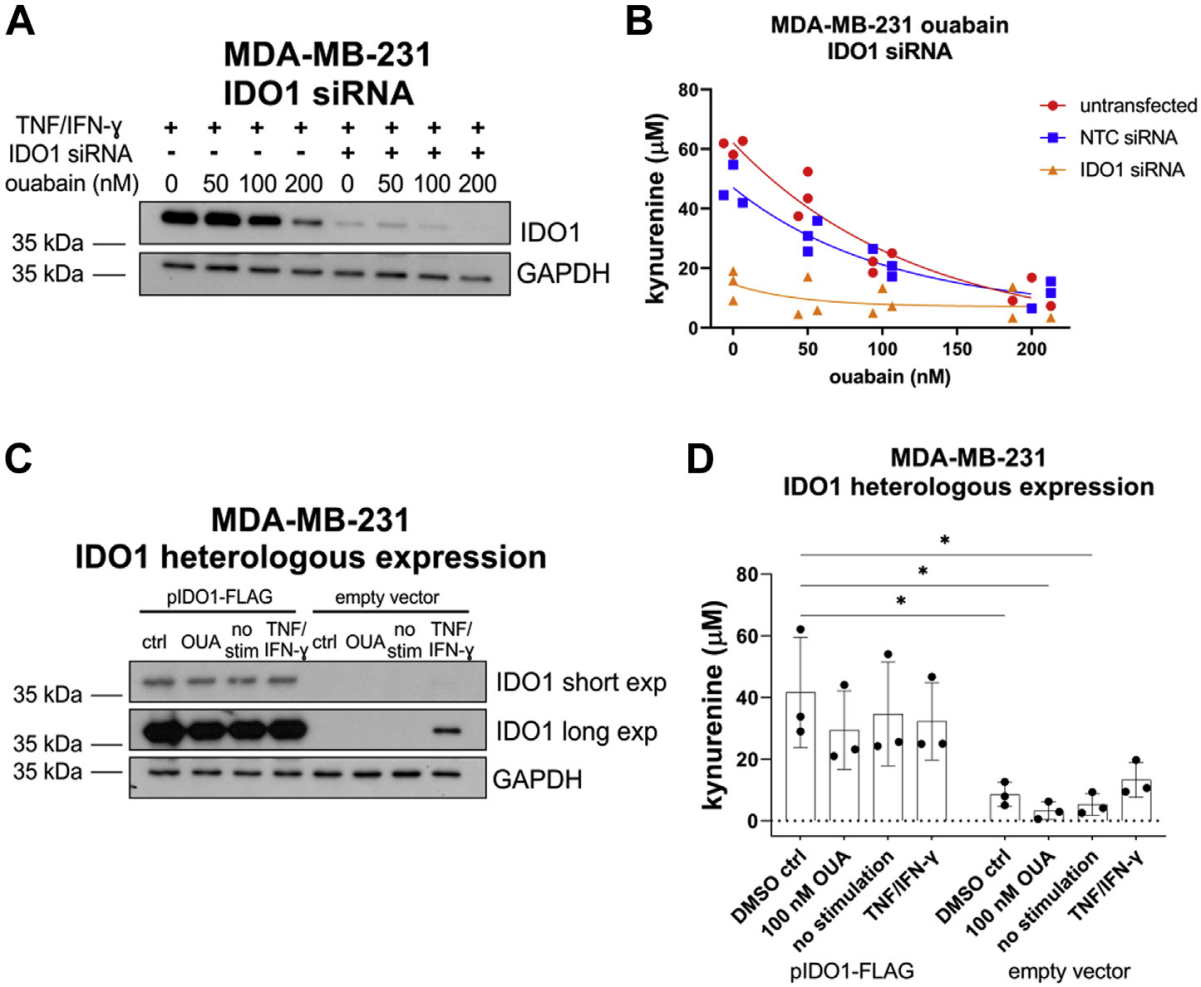
**IDO1 expression is sufficient to produce kynurenine in MDA-MB-231 cells.***A*, representative Western blot of IDO1 protein expression in TNF/IFN-ɣ-stimulated MDA-MB-231 cells, pretreated with increasing concentrations of ouabain or 0.05% DMSO, and transfected with an IDO1 targeting siRNA pool or nontargeting control (NTC) siRNA pool. *B*, kynurenine assay of TNF/IFN-ɣ-stimulated MDA-MB-231 cells, pretreated with increasing concentrations of ouabain or 0.05% DMSO, and transfected with an IDO1 targeting siRNA pool or nontargeting control (NTC) siRNA pool. Three independent experiments. *C*, representative Western blot of IDO1 protein expression in MDA-MB-231 cells transfected with pIDO1-FLAG or empty vector and pretreated with 0.05% DMSO (ctrl), 100 nM ouabain (OUA), no stimulation (no stim), or TNF/IFN-ɣ. *D*, kynurenine assay of MDA-MB-231 cells transfected with pIDO1-FLAG or empty vector and pretreated with 0.05% DMSO or 100 nM ouabain or no stimulation or TNF/IFN-ɣ. Three independent experiments. ∗*p* < 0.05, one-way ANOVA Tukey’s post-hoc. IDO1, indoleamine-pyrrole 2′,3′-dioxygenase 1.

In addition to IDO1, several enzymes are integral to the tryptophan/kynurenine metabolic pathway (25). Therefore, we tested whether IDO1 is the primary determinant of kynurenine production, focusing on MDA-MB-231 cells. We confirmed by Western blot that IDO1 protein expression can be knocked down with siRNA in TNF/IFN-γ-stimulated MDA-MB-231 cells pretreated with different concentrations of ouabain (Fig. 3*A*). Kynurenine assays showed that IDO1 depletion resulted in a drastic reduction in kynurenine production by these cells, from 47 ± 7 μM kynurenine in control cells to 15 ± 5 μM kynurenine in cells transfected with IDO1-targeting siRNA. Importantly, ouabain treatment resulted in a modest downregulation of IDO1 expression in IDO1 siRNA-transfected cells (Fig. 3*A*) but did not have a statistically significant effect on kynurenine production (Fig. 3*B*).

Having shown that IDO1 is necessary for kynurenine production in TNF/IFN-γ-stimulated cells, we tested whether IDO1 was sufficient in driving kynurenine production in the absence or presence of cytokines. In agreement with previous reports (30), we observed that TNF/IFN-γ stimulates IDO1 expression and kynurenine production (Fig. 3*C*). In empty vector-transfected cells, IDO1 was not observed by Western blot unless stimulated with TNF/IFN-γ (Fig. 3*C*, IDO1 long exposure). Heterologous expression of IDO1 (pIDO1-FLAG) resulted in intense overexpression of IDO1 regardless of cytokine stimulation or pretreatment with 100 nM ouabain (Fig. 3*C*, IDO1 short exposure). Kynurenine assays of pIDO1-FLAG transfected cells showed that heterologous expression of IDO1 was sufficient to significantly increase kynurenine concentrations in the media of transfected cells, above those of empty vector-transfected cells (Fig. 3*D*). This demonstrated that in TNF/IFN-γ-stimulated cells, IDO1 is the major determinant of kynurenine production and that ouabain does not affect kynurenine production independently of IDO1. Importantly, it also showed that ouabain did not affect levels of exogenously expressed IDO1. In combination with the observation that ouabain affects both mRNA and protein levels of endogenous IDO1, this suggested that the mechanism of IDO1 regulation is likely to transcriptional or posttranscriptional.

### ATP1A1 knockdown synergistically enhances the effect of cardiac glycosides on IDO1 expression and function

We aimed to validate that cardiac glycosides were impacting immune checkpoints through their primary target by performing siRNA knockdown of ATP1A1, the ɑ1 subunit of NKA. NKA promotes maintenance of a stable cell membrane potential by conducting sodium export (3 Na^+^) and potassium import (2 K^+^), each against their concentration gradients (35). NKA is a transmembrane protein consisting of three subunits: α, β, and γ, each with different isoforms expressed in a cell-type-dependent manner (36, 37, 38). The α subunit is the pore-forming subunit and contains the ATP-binding catalytic domain on the cytoplasmic side (37). There are four isoforms of the α subunit encoded by genes *ATP1A1* (α1), *ATP1A2* (α2), *ATP1A3* (α3), and *ATP1A4* (α4), α1 being the most widely expressed across different tissues (36, 37, 39). The α subunit is a target of most NKA inhibitors, including cardiac glycosides, a well-characterized class of NKA functional inhibitors including ouabain, digoxin, digitoxin, and bufalin, among others (36, 40, 41, 42, 43).

**Figure 4:**
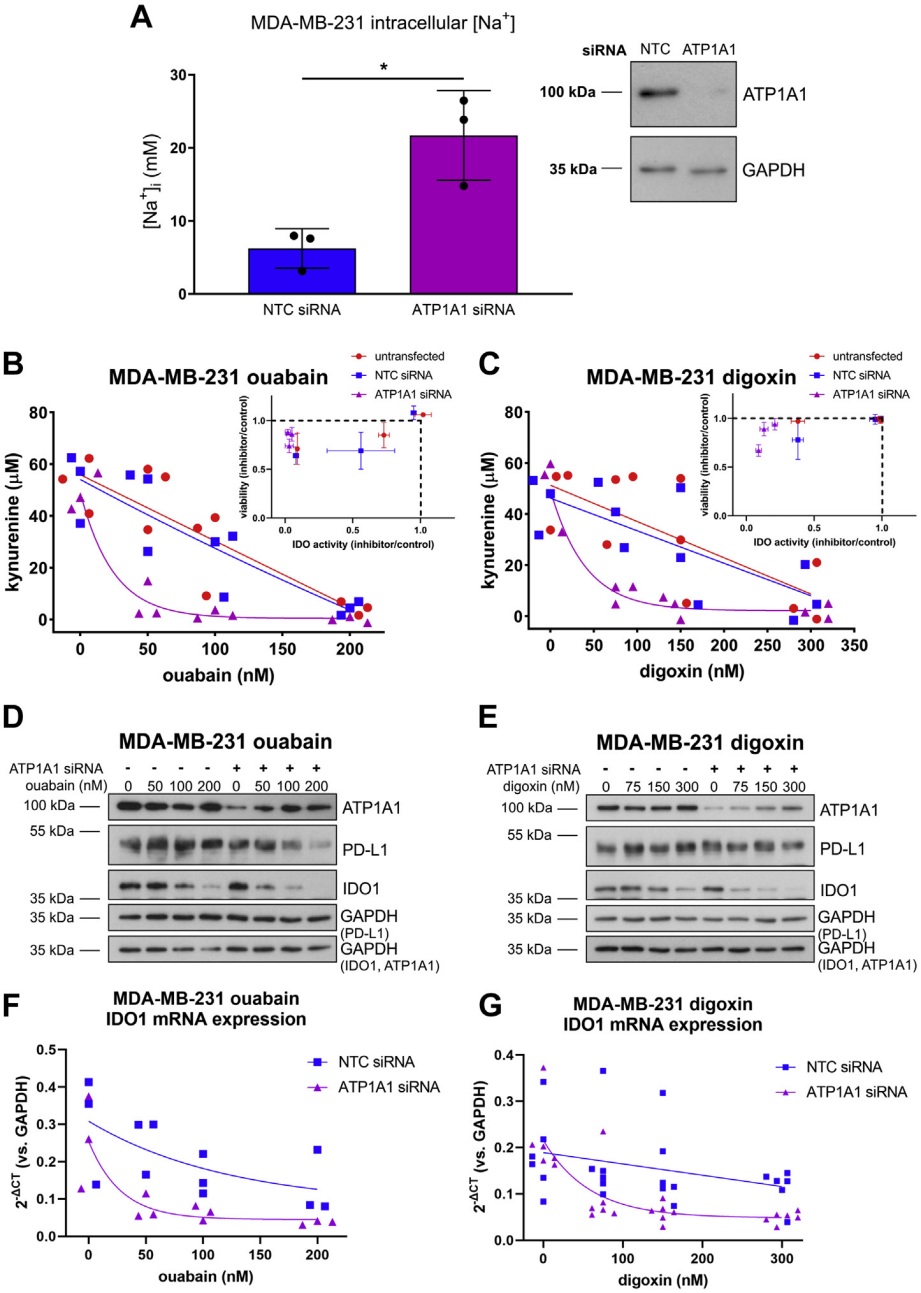
**ATP1A1 knockdown and cardiac glycoside treatment result in synergistic inhibition of IDO1 expression and activity in MDA-MB-231 cells.***A*, SBFI intracellular Na^+^ measurements in NTC *versus* ATP1A1 siRNA transfected cells, n = 3. ∗*p* < 0.05, unpaired, two-tailed *t* test. *Inset*, representative Western blot of ATP1A1 and GAPDH protein expression in samples transfected with NTC or ATP1A1 siRNA. *B*, kynurenine assay of untransfected, NTC siRNA, or ATP1A1 siRNA-transfected TNF/IFN-ɣ (24 h) stimulated MDA-MB-231 cells treated with 0.05% DMSO or increasing concentrations of ouabain (50, 100, 200 nM), n = 3. *Inset*, normalized viability (inhibitor/DMSO control) *versus* normalized IDO activity (inhibitor/DMSO control) from each of three wells of one kynurenine assay. *C*, kynurenine assay of untransfected, NTC siRNA, or ATP1A1 siRNA transfected TNF/IFN-ɣ (24 h) stimulated MDA-MB-231 cells treated with 0.05% DMSO or increasing concentrations of digoxin (75, 150, 300 nM), n = 3. *Inset*, normalized viability (inhibitor/DMSO control) *versus* normalized IDO activity (inhibitor/DMSO control) from each of three wells of one kynurenine assay. *D*, representative Western blot of ATP1A1, PD-L1, IDO1, and GAPDH protein expression for experiments in (*B*). The same samples were run and blotted simultaneously on two separate membranes. GAPDH loading control for each blot is noted. *E*, representative Western blot of ATP1A1, PD-L1, IDO1, and GAPDH protein expression for experiments in (*C*). The same samples were run and blotted simultaneously on two separate membranes. GAPDH loading control for each blot is noted. *F*, IDO1 mRNA expression for NTC or ATP1A1 siRNA transfected, TNF/IFN-ɣ (24 h) stimulated MDA-MB-231 cells treated with increasing concentrations of ouabain, n = 3. *G*, IDO1 mRNA expression for NTC or ATP1A1 siRNA transfected, TNF/IFN-ɣ (24 h) stimulated MDA-MB-231 cells treated with increasing concentrations of digoxin, n = 3. IDO1, indoleamine-pyrrole 2′,3′-dioxygenase 1; SBFI, sodium-binding benzofuran isophthalate.

To explore the role of NKA in IDO1 regulation, we performed siRNA-mediated knockdown of ATP1A1. This resulted in increased levels of intracellular Na^+^, indicating functionally relevant levels of knockdown (Fig. 4*A*). However, we noted that the increase in intracellular Na^+^ due to ATP1A1 knockdown was modest compared with that observed with treatment with 100 nM ouabain and only reached levels at which we did not observe inhibition of kynurenine production in ouabain-treated cells (around 20 nM, Fig. 2*D*). Indeed, ATP1A1 siRNA knockdown by itself did not decrease kynurenine production in MDA-MB-231 cells (Fig. 4, *B* and *C*). Decreased kynurenine was only observed when ATP1A1 siRNA-transfected cells were also treated with ouabain or digoxin, and this decrease was cardiac glycoside concentration dependent (Fig. 4, *B* and *C*). ATP1A1 knockdown was confirmed by Western blot (Fig. 4, *D* and *E*). While increasing concentrations of cardiac glycoside treatment upregulated ATP1A1 expression in siRNA-transfected cells, there remained less ATP1A1 in each condition compared with nontargeting control (NTC) siRNA-transfected cells (Fig. 4, *D* and *E*). Independent of ion transport activity and corresponding changes in intracellular ion concentrations, certain concentrations of cardiac glycosides and NKA have been implicated in growth pathways regulating cell adhesion, motility, and proliferation such as PI3K/Akt and Ras/MAPK (44, 45, 46, 47). We observed decreased percent live cells with ATP1A1 siRNA transfection and cardiac glycoside treatment above 100 nM ouabain (Fig. 4, *B* and *C* insets). Digoxin treatment was less cytotoxic (Fig. 4*C*, inset) than ouabain (Fig. 4*B*, inset) at corresponding concentrations.

Ouabain and digoxin treatment of MDA-MB-231 cells decreased IDO1 protein expression in a concentration-dependent manner in both control and ATP1A1-depleted cells (Fig. 4, *D* and *E*). Decreased IDO1 also occurred at the mRNA level in ouabain- and digoxin-treated cells (Fig. 4, *F* and *G*). Furthermore, the cardiac glycoside-mediated suppression of IDO1 protein and mRNA levels and kynurenine production were drastically enhanced in ATP1A1 siRNA-transfected cells (Fig. 4, *B*–*G*). Of note, we also measured levels of PD-L1, the expression of which was inhibited in ouabain- but not digoxin-treated MDA-MB-231 cells (Fig. 4, *D* and *E*), and only the decrease in PD-L1 protein expression due to ouabain was enhanced by ATP1A1 knockdown (Fig. 4*D*).

**Figure 5:**
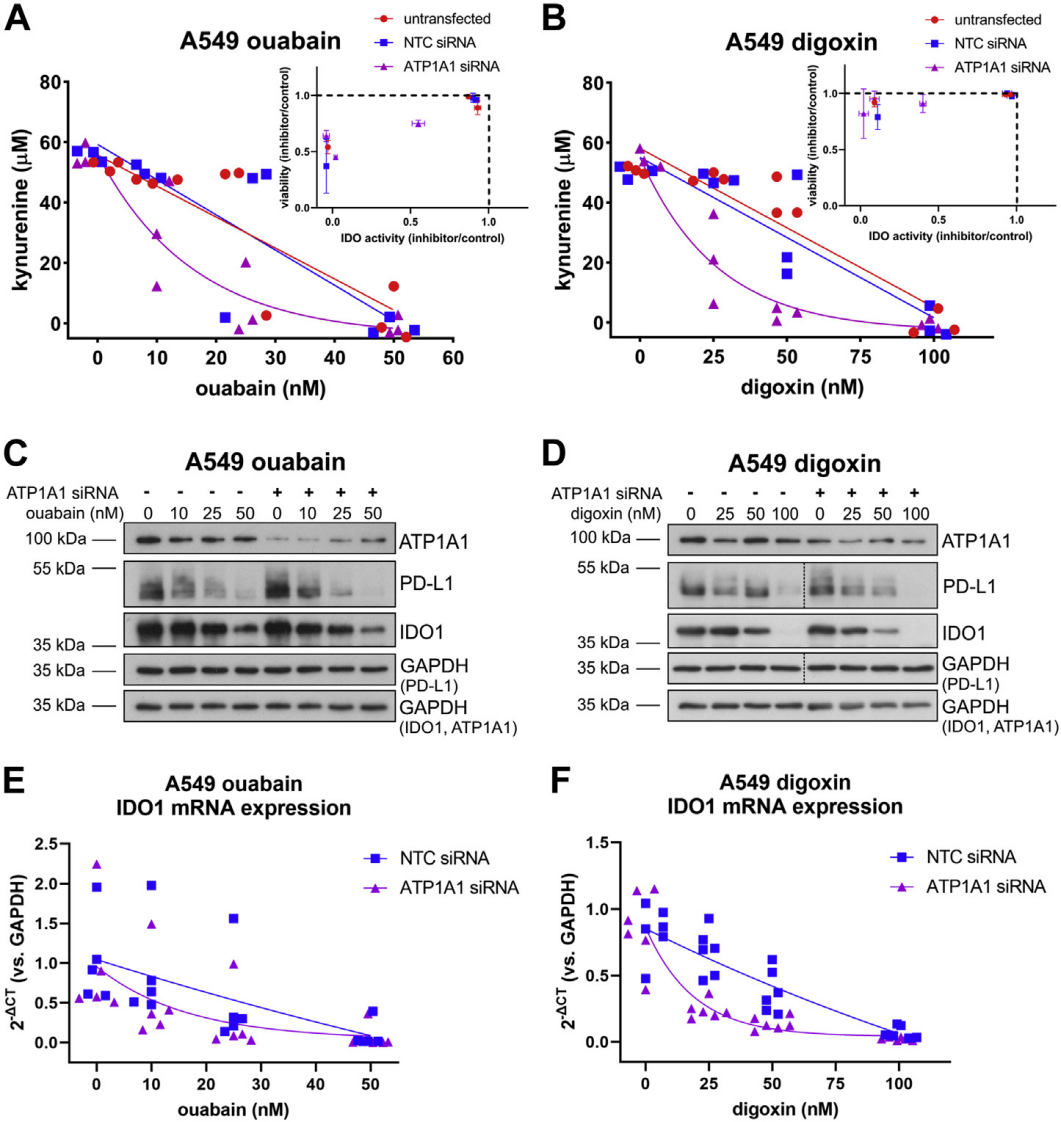
**ATP1A1 knockdown and cardiac glycoside treatment result in synergistic inhibition of IDO1 expression and activity in lung cancer A549 cells.***A*, kynurenine assay of untransfected, NTC siRNA, or ATP1A1 siRNA transfected TNF/IFN-ɣ (24 h) stimulated A549 cells treated with 0.05% DMSO or increasing concentrations of ouabain (10, 25, 50 nM), n = 3. (Inset normalized viability (inhibitor/DMSO control) *versus* normalized IDO activity (inhibitor/DMSO control) from each of three wells of one kynurenine assay). *B*, kynurenine assay of untransfected, NTC siRNA, or ATP1A1 siRNA transfected TNF/IFN-ɣ (24 h) stimulated A549 cells treated with 0.05% DMSO or increasing concentrations of digoxin (25, 50, 100 nM), n = 3. *Inset*, normalized viability (inhibitor/DMSO control) *versus* normalized IDO activity (inhibitor/DMSO control) from each of three wells of one kynurenine assay. *C*, representative Western blot of ATP1A1, PD-L1, IDO1, and GAPDH protein expression for experiments in *A*. The same samples were run and blotted simultaneously on two separate membranes. GAPDH loading control for each blot is noted. *D*, representative Western blot of ATP1A1, PD-L1, IDO1, and GAPDH protein expression for experiments in *B*. The same samples were run and blotted simultaneously on two separate membranes. GAPDH loading control for each blot is noted. The *dashed vertical lines* in PD-L1 and accompanying GAPDH panels indicate an empty lane that has been spliced out. *E*, IDO1 mRNA expression for NTC or ATP1A1 siRNA transfected, TNF/IFN-ɣ (24 h) stimulated A549 cells treated with increasing concentrations of ouabain, n = 3. *F*, IDO1 mRNA expression for NTC or ATP1A1 siRNA transfected, TNF/IFN-ɣ (24 h) stimulated A549 cells treated with increasing concentrations of digoxin, n = 3. IDO1, indoleamine-pyrrole 2′,3′-dioxygenase 1.

To see whether the effects of combining cardiac glycoside treatment with depletion of ATP1A1 were cell-type-dependent, we performed equivalent experiments in TNF/IFN-ɣ (24 h) stimulated A549 cells (Fig. 5). In this cell line, ATP1A1 siRNA knockdown and cardiac glycoside treatment produced a similar synergistic effect on kynurenine production as in MDA-MB-231 cells (Fig. 5, *A* and *B*). ATP1A1 knockdown was confirmed by Western blot (Fig. 5, *C* and *D*). In A549 cells, we also observed that increasing concentrations of cardiac glycoside treatment upregulated ATP1A1 expression in ATP1A1 siRNA-transfected cells (Fig. 5, *C* and *D*). However, there remained less ATP1A1 in each condition compared with NTC siRNA-transfected cells (Fig. 5, *C* and *D*). ATP1A1 siRNA knockdown by itself did not decrease kynurenine production in A549 cells (Fig. 5, *A* and *B*). Decreased kynurenine was only observed when ATP1A1 siRNA-transfected cells were also treated with ouabain or digoxin, and this decrease was cardiac glycoside concentration dependent (Fig. 5, *A* and *B*). Again, we observed drastic decreases in kynurenine levels in cells transfected with ATP1A1 siRNA and treated cardiac glycosides at concentrations that did not affect cell viability (Fig. 5, *A* and *B* insets), particularly for digoxin treatment (*e.g.*, almost complete loss of kynurenine corresponding to over 80% viability).

As in the case of MDA-MB-231 cells, ouabain and digoxin treatment of A549 cells decreased IDO1 protein expression in a concentration-dependent manner (Fig. 5, *C* and *D*). Decreased IDO1 was reflected at the mRNA level in ouabain- and digoxin-treated cells, albeit with more variability in the ouabain-treated cells (Fig. 5*C*). Furthermore, ATP1A1 siRNA knockdown enhanced the effect of cardiac glycoside treatment. Increasing concentrations of cardiac glycosides caused more inhibition of IDO1 expression in ATP1A1 siRNA-treated cells compared with NTC siRNA-transfected cells (Fig. 5, *C* and *D*). The effect of ATP1A1 knockdown and ouabain treatment on IDO1 protein expression in A549 cells (Fig. 5*C*) was less profound compared with the same condition in MDA-MB-231 cells (Fig. 4*C*). In contrast to MDA-MB-231 cells, PD-L1 expression was inhibited in ouabain- and digoxin-treated A549 cells independent of ATP1A1 knockdown (Fig. 5, *C* and *D*).

### Cardiac glycosides inhibit STAT1 phosphorylation

To gain further mechanistic insight, we explored potential signalling pathways that could mediate the effects of cardiac glycosides and ATP1A1 knockdown on immune checkpoint protein expression in TNF/IFN-ɣ-stimulated cancer cells. The IFN-γ receptor is a dimeric molecule, which becomes activated upon ligand binding, triggering Janus Kinase/Signal Transducer and Activator of Transcription (JAK/STAT) signalling and thus, downstream gene expression. STAT1 has been associated with IDO1 gene expression (29, 31).

**Figure 6:**
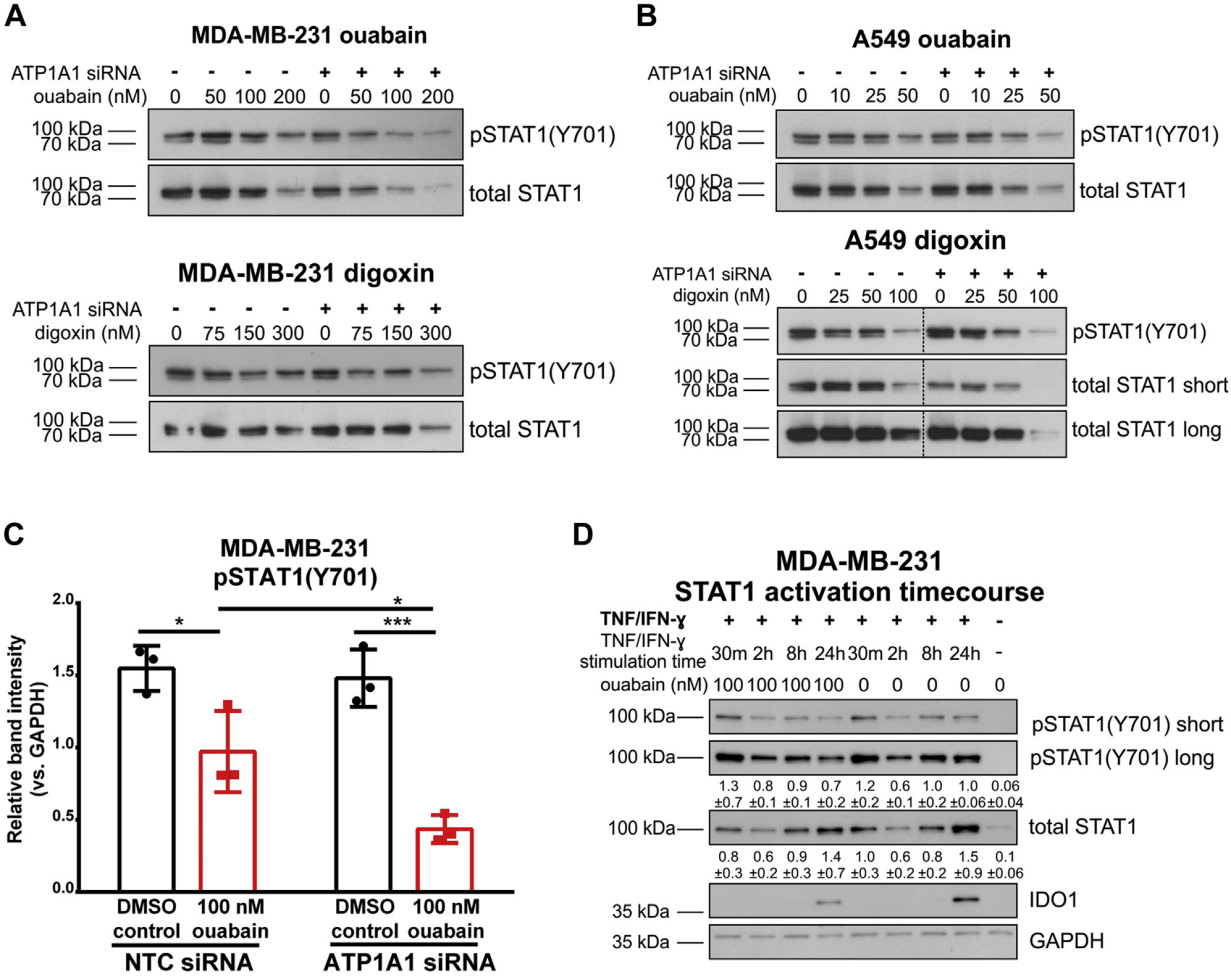
**ATP1A1 knockdown enhances cardiac glycoside-mediated inhibition of STAT1(Y701) phosphorylation.***A*, representative levels of pSTAT1(Y701) and total STAT1 are shown for the MDA-MB-231 ouabain and digoxin experiments in Figure 4. For loading controls, see Figure 4, *D* and *E*. *B*, representative levels of pSTAT1(Y701) and total STAT1 are shown for the A549 ouabain and digoxin experiments in Figure 5. For loading controls, see Figure 5, *C* and *D*. The *dashed vertical lines* indicate one lane (ladder) that has been spliced out. *C*, quantification of pSTAT1(Y701) in western blots of NTC or ATP1A1 siRNA transfected TNF/IFN-ɣ (24 h) stimulated MDA-MB-231 cells treated with 100 nM ouabain. ∗*p* < 0.05, ∗∗∗*p* < 0.001, Two-factor ANOVA, Tukey’s multiple comparisons. *D*, STAT1 activation time-course experiment in MDA-MB-231 cells treated with 100 nM ouabain or 0.05% DMSO and stimulated for 30 min, 2 h, 8 h, or 24 h with TNF/IFN-γ. Mean intensities ±standard deviation, measured by densitometry of blots from three independent time-course experiments, are listed under the pSTAT1(Y701) long and total STAT1 panels.

We probed for STAT1 phosphorylated at the C-terminal tyrosine residue Tyr-701 (pSTAT1(Y701)) in TNF/IFN-ɣ (24 h) stimulated MDA-MB-231 and A549 cells, which had been transfected with NTC or ATP1A1 siRNA and treated with ouabain or digoxin. Levels of pSTAT1 decreased with increasing concentrations of cardiac glycosides and ATP1A1 knockdown enhanced the effect of cardiac glycoside treatment (Fig. 6). Phosphorylation of STAT1 was inhibited at the highest concentrations of cardiac glycosides in both cell lines: 200 nM ouabain in MDA-MB-231 (Fig. 6*A*, upper blot), 300 nM digoxin in MDA-MB-231 (Fig. 6*A*, lower blot), 50 nM ouabain in A549 (Fig. 6*B*, upper blot), and 100 nM digoxin in A549 (Fig. 6*B*, lower blot). In the presence of ATP1A1 siRNA, phosphorylation of STAT1(Y701) was inhibited at lower concentrations of cardiac glycosides. Decreased STAT1 activation was more prominent at 50 nM ouabain and 75 nM digoxin in MDA-MB-231 (Fig. 6*A*) and 10 to 25 nM ouabain in A549 (Fig. 6*B*, upper blot). In contrast, STAT1 activation was similar with digoxin treatment of A549 with and without ATP1A1 siRNA (Fig. 6*B*, lower blot). pSTAT1(Y701) was significantly inhibited by 100 nM ouabain in MDA-MB-231 cells (Fig. 6*C*).

We then performed time-course experiments to further explore the potential mechanism employed by cardiac glycosides to modulate STAT1 activation. In all experimental conditions, total STAT1 expression followed a similar pattern to pSTAT1(Y701), which is in agreement with previous reports demonstrating that Tyr-701 phosphorylation stabilizes STAT1 protein (48). Interestingly, we did not observe any effects of cardiac glycosides on the early activation of STAT1 following cytokine treatment. Rather, the inhibitory effect of 100 nM ouabain on pSTAT1(Y701) was observed after 24-h stimulation, which corresponds with the timeframe of IDO1 protein expression in response to TNF/IFN-γ stimulation (Fig. 6*D*). These findings demonstrated that IDO1 upregulation occurs at the later stages of JAK/STAT activation (24 h). Cardiac glycoside treatment did not affect the initiation or peak activity (observed at 30 min) of JAK-STAT signaling, but it affected the length of signaling by curtailing the later stages of activation, leading to suppression of IDO1 expression.

## Discussion

We investigated the role of ion transport in immune checkpoint protein function and expression in cancer cells. From a screen of commonly used ion transport targeting small molecules, we found cardiac glycosides to be novel inhibitors of the IDO1 pathway in MDA-MB-231 and A549 cancer cells. Cardiac glycosides ouabain and digoxin potently inhibited kynurenine production and IDO1 expression in both cell lines. Knockdown of the primary target of cardiac glycosides, ATP1A1, enhanced the cardiac glycoside-mediated inhibition of IDO1 expression and kynurenine production in both cell lines. Our data suggest a potential role for the ion transport machinery in the interactions between cancer and immune cells.

In our screen of 31 ion transport targeting compounds, celecoxib, diclofenac, and ouabain had a significant effect on kynurenine production. Kynurenine levels depend on flux through the entire tryptophan metabolic pathway, particularly at the transformation of kynurenine to anthranilic acid catalyzed by kynureninase (KYNU). KYNU has been reported to be responsive to IFN-γ (49), although this is not always the case in cancer cells (50). It would be interesting to explore in the future the effects of inflammatory cytokine stimulation on KYNU and other enzymes in the tryptophan metabolic pathway. Additionally, kynurenine can be reimported by large neutral amino acid transporters (51, 52, 53). Therefore, it cannot always be expected to observe a linear relationship between tryptophan metabolism and the kynurenine concentration measured in supernatants. This could explain why the screen did not reveal other potentially more subtle regulators of IDO1. Ouabain was the only compound whose effect on IDO1 had not been previously reported. Its effect on IDO1 could be related to the corresponding changes in intracellular Na^+^ or non-Na^+^ related signaling by NKA, a ubiquitous ion transport and signaling protein, or steroid receptor complex interaction (43). With respect to Na^+^, ouabain increases the concentration of intracellular Na^+^ by inhibiting NKA in a dose-dependent manner, and kynurenine levels are inversely proportional to Na^+^ levels in ouabain-treated cells (Fig. 2*C*) (54). Similarly, partial knockdown of ATP1A1 only causes a modest increase in Na^+^ levels without affecting kynurenine production, as is the case in cells treated with low doses of ouabain. However, we note that no drugs impacting intracellular Na^+^ by other mechanisms such as through voltage-gated sodium channels (VGSCs) were hits in the screen. A549 cells lack functional VGSCs, so we expect drugs targeting these to have limited effect (55, 56). However, functional VGSCs are expressed in MDA-MB-231 cells (3). Veratridine activates VGSCs and increases intracellular Na^+^, so one might expect a similar effect on IDO1 as ouabain (54, 57). It is possible that VGSCs in MDA-MB-231 cells subject to our experimental conditions are not active or expressed enough to affect IDO1. Furthermore, these molecules affect passive Na^+^ transport. With chronic drug treatment of cells, VGSC expression may be equilibrated such that intracellular Na^+^ remains relatively constant and/or elevated intracellular Na^+^ allosterically upregulates NKA activity to send levels back to “normal.” While there is evidence that IFN-ɣ can inhibit NKA expression, we observed no IFN-ɣ-dependent decrease in NKA expression or increase in intracellular Na^+^ in our experiments (58).

Ouabain was generally more potent than digoxin in our experiments, although depending on conditions they have had either the same potency (59) or ouabain is more potent than digoxin (60, 61, 62). Ouabain and digoxin are cardenolides consisting of a glycosylated steroid core—L-rhamnose (ouabain) or trisaccharide (digoxin)—and a five-membered lactone ring. Binding properties of the cardiac glycosides to the alpha subunit and isoform selectivity (ouabain ɑ1 ∼ ɑ2 ∼ ɑ3, digoxin ɑ2/3 > ɑ1) are largely determined by a hydrogen bond network between the steroid core and polar side chains of transmembrane helices M1, M2, and M6 (59, 62, 63, 64, 65). Thus, differences in potency may arise from the inherent molecular structure as well as the subunit isoform distribution in the cells or solution of purified NKA being tested.

In our experiments, ATP1A1 knockdown alone was insufficient to inhibit IDO1, but drastically enhanced IDO1 inhibition by cardiac glycosides. ATP1A1 knockdown increased intracellular Na^+^ levels, albeit to a much lower extent than that observed following cardiac glycoside treatment. This suggests that the ATP1A1 knockdown was only partial, allowing for sufficient NKA activity to remain. Subsequent cardiac glycoside inhibitor treatment may compromise the remaining NKA and trigger a strong cellular response even at low concentrations. We observed a similar synergistic effect of cardiac glycosides and ATP1A1 knockdown on STAT1 phosphorylation at site Y701, suggesting the JAK/STAT pathway as a mediator between cardiac glycosides and immune checkpoint proteins. This indicates that the drug (cardiac glycoside) to target (ATP1A1) ratio is critical for maximum suppression of IDO1 by cardiac glycosides.

Growth factors (EGF, PDGF, G-CSF) and proinflammatory cytokines (IFN-γ, IL-2, IL-6) can activate the JAK/STAT pathway (66, 67, 68, 69, 70, 71). IL-2 has also been shown to activate NKA and upregulate its expression in T cells, and ouabain inhibits T cell activation *via* IL-2 signaling (72, 73, 74). Furthermore, in cardiac myocytes and epithelial cells, digoxin activates cell growth pathways independent of NKA ion transport function and changes in ion concentration (43, 75, 76). In cancer cell lines, ouabain activates Src kinase and PI3K/Akt/mTOR; Src kinase mediates activation of EGFR and downstream Ras/MAPK signaling (77, 78, 79, 80, 81, 82, 83, 84, 85). However, it remains controversial whether these signaling mechanisms involve direct NKA-Src kinase interaction or alteration of ATP/ADP concentrations *via* inhibition of NKA activity (77, 79, 85, 86, 87, 88, 89). Src kinase has also been shown to activate STAT1 and STAT3 (71, 90). This is inconsistent with our observation that STAT1 activation is inhibited by ouabain (Fig. 5), suggesting that NKA inhibition in our experimental conditions may inhibit STAT1 in a manner independent of Src kinase or EGFR.

Of note, cardiac glycosides have been shown to regulate immune signaling. Previous reports have shown that bufalin inhibits Type I interferon signaling (IFN-β)-induced gene expression through inhibition of RNA helicase RIG-I ATPase activity (91). In the same paper, STAT1 induction was also reduced with ATP1A1 knockdown in mouse embryonic fibroblasts (91). In this context, our findings reveal cardiac glycoside-mediated inhibition of the Type II interferon (IFN-γ) response through the JAK/STAT pathway. Importantly, we show that although the initial activation of the JAK/STAT pathway is unaffected, the duration of activation, as measured by levels of phosphorylation and total protein levels of STAT1, is impaired by cardiac glycosides. There are multiple negative regulators of STAT1 levels and phosphorylation, including cytoplasmic phosphatases (*e.g.*, SHP1/2, PTP1B) (92), components of the linear ubiquitination complex (*e.g.*, HOIP) (93), and the JAK/STAT-induced family of suppressors of cytokine signaling (SOCS) proteins (94). Future studies should explore the effect of cardiac glycosides on these families of STAT1 regulators to gain more understanding into the potential of cardiac glycosides to regulate immune cell function through their primary target ATP1A1, downstream effector STAT1, and immune checkpoint proteins in cancer cells. Overall, exploring the role of plasma membrane ion transport in cancer immunity could help us understand responses to immune checkpoint blockade in individuals taking ion transport modifying drugs (such as cardiac glycosides) and potentially enable repurposing of currently used drugs for adjunct immunotherapies. This will inform our understanding of the potential risks or benefits associated with patients taking certain drugs for other indications should they require cancer treatment. Finally, such studies would further elucidate the role of ATP1A1, the expression of which is reduced in prostate, breast, and kidney cancer and associated with poorer survival of kidney cancer patients (95). In this respect, the link we establish between cardiac glycoside treatment and immune checkpoint protein expression in cancer cells is likely to have far-reaching implications.

## Experimental procedures

### Reagents

Human recombinant IFN-ɣ (300-02) was obtained from PeproTech, and TNF (210-TA-020) was purchased from R&D systems. (L-)kynurenine (K8625), ouabain octahydrate (O3125), digoxin (D6003), p-dimethylaminobenzaldehyde (D2004), and gramicidin (G5002) were purchased from Sigma Aldrich. SBFI-AM (sc-215841) was purchased from Santa Cruz Biotechnology. Trichloroacetic acid (11462691) and glacial acetic acid (010994-AC) were purchased from Fisher Scientific. Cell culture grade DMSO (A3672.0100) was purchased from AppliChem. Pluronic F-127 (59004) was purchased from Biotium.

### Cell culture

Human triple-negative metastatic breast cancer MDA-MB-231 cells were a generous gift from Mustafa Djamgoz. Human adenocarcinoma alveolar basal epithelial lung cancer A549 cell line was a gift from Tyson Sharp. All experiments with MDA-MB-231 and A549 cells were cultured at 37 °C, 5% CO_2_ in Dulbecco’s Modified Eagle’s Medium (DMEM) (21969035, Gibco) supplemented with 10% heat-inactivated Fetal Calf Serum (FCS), 1% L-glutamine (25030081, Gibco), and 1% penicillin/streptomycin (15070063, Gibco). Both cell lines tested negative for *mycoplasma* by PCR (J66117, Alfa Aesar).

### Ion channel targeting small-molecule treatment and screen

In total, 50,000 MDA-MB-231 or 10,000 A549 cells per well were seeded in clear 96-well plates. The next day, media was exchanged, and cells were pretreated with ion channel compounds at the specified final concentration for 24 h (Table S1). Cells were stimulated with TNF and IFN-ɣ and treated with ion channel compounds or DMSO. Throughout, cells were treated with 1 U/ml IFN-ɣ and 6.25 ng/ml TNF. For the small-molecule screen (Figs. 1, *C* and *D* and 2, *A* and *B*) 1 U/ml IFN-ɣ and 25 ng/ml TNF were used. After 24 h, the kynurenine assay was performed. Kynurenine data were compared with their respective DMSO control, either 1% or 0.05%.

### Kynurenine assay

Kynurenine concentrations of cell supernatants were determined as described previously (30), using kynurenine dissolved in DMEM 10% FCS as standards. Kynurenine standards stock was 50 mM in 0.5 M HCl. First, 150 μl cell supernatant was collected from treated cells and transferred to a 96-well round-bottom plate. In total, 10 μl 30% trichloroacetic acid was added to each well and the plate incubated at 50 °C for 30 min. The plate was centrifuged at 800*g* for 10 min at room temperature. In total, 100 μl supernatant was transferred to a 96-well flat-bottom plate; 100 μl 1.2% w/v p-dimethylaminobenzaldehyde in acetic acid was added to each well and the plate incubated for 10 min at room temperature. Absorbance at 492 nm was measured on a VersaMax microplate reader (Molecular Devices). IC50 for ouabain and digoxin to inhibit kynurenine production was calculated by a nonlinear fit (variable slope, four parameters). When viability was measured, cells were lifted with 0.05% Trypsin-EDTA (25300054, Gibco), resuspended in PBS, stained with trypan blue (T10282, Gibco), and counted using the Countess II Automated Cell Counter (Thermo Fisher Scientific).

### RNA interference and overexpression

ON-TARGETplus small interfering RNA (siRNA) SMARTpool oligonucleotides were purchased from Horizon Discovery (ATP1A1 L-006111-00-0005, IDO1 L-010337-01-0005, NTC D-001810-10-05). In total, 200,000 A549 or 250,000 MDA-MB-231 cells per well were seeded in 12-well plates 1 day before transfection. Cells were transfected with a total of 25 nM siRNA using TransIT-siQuest transfection reagent (MIR 2114, Mirus Bio) and Opti-MEM medium (31985070, Gibco) for 5 h before being replaced with complete medium. After 5 h, cells were washed with PBS and treated with or without cardiac glycosides in 0.05% DMSO-containing complete medium. Unless otherwise noted, after 36 h of drug pretreatment, cells were washed with PBS and stimulated with 6.25 ng/ml TNF and 1 U/ml IFN-ɣ with or without cardiac glycosides in 0.05% DMSO complete medium. Cells were assayed after 24 h of cytokine stimulation. (5 days in culture).

A pCMV3-C-Flag-IDO1 (NM_002164.4) (SinoBiological, HG11650-CF) and a pCMV3-C-Flag empty vector (CV012) were purchased from SinoBiological. In total, 100,000 MDA-MB-231 cells/well were seeded in a 12-well plate 1 day before transfection. DNA transfections were carried out using JetPrime transfection reagent (Polyplus, 114-01). 1 μg DNA and transfection buffer were first mixed, then transfection reagent was added to keep a 2:1 v/w ratio with DNA. The total reaction volume per sample was 75 μl. Following a 10-min RT incubation, the reaction mix was added to the cells growing in complete growth media. The transfection reaction was allowed to proceed for at least 4 h. Cells were then washed with PBS and left overnight in complete growth media. On the next day, either 100 nM ouabain in 0.05% DMSO or 6.25 ng/ml TNF and 1 U/ml IFN-ɣ stimulation was added for 24 h. A 0.05% DMSO control and a no stimulation control (media only) were also set up. The kynurenine assay and the Western blot sample collection were carried out 24 h post drug/cytokine treatment (4 days in culture).

### qRT-PCR

Total RNA was extracted with QIAzol (79306, Qiagen), and RNeasy Mini kit (74104, Qiagen). cDNA was synthesized by random hexamers using Superscript III reverse transcriptase (18080093, Invitrogen). qRT-PCR of ATP1A1, IDO1, PD-L1, and GAPDH was performed using Fast SYBR Green qRT-PCR (4385610, Applied Biosystems). Human ATP1A1 was quantified using a Quantitect Primer Assay (249900, GeneGlobe ID QT00059962, Qiagen). Human IDO1, PD-L1, and GAPDH were quantified using the following forward and reverse primers (Sigma Aldrich): IDO1 Forward, 5′-GGCTTTGCTCTGCCAAATCC-3′, IDO1 Reverse 5′-TTCTCAACTCTTTCTCGAAGCTG-3′, PD-L1 Forward 5′-CATCTTATTATGCCTTGGTGTAGCA-3′, PD-L1 Reverse 5′-GGATTACGTCTCCTCCAAATGTG-3′, GAPDH Forward 5′-GGAGTCAACGGATTTGGTCGTA-3′, GAPDH Reverse 5′-GGCAACAATATCCACTTTACCAGAGT-3′. Relative mRNA levels were calculated using the ΔΔC_T_ method. mRNA levels were normalized to GAPDH.

### Western blot

Cells were washed once with PBS and lysed with ice-cold cell lysis buffer (5 mM EDTA, 150 mM NaCl, 10 nM Tris-HCl, pH 7.2, 0.1% SDS, 0.1% Triton X-100, and 1% sodium deoxycholate) containing protease and phosphatase inhibitor mixtures P8340, P5726, and P0044 (Sigma). Protein concentration was determined by Pierce bicinchoninic acid assay (23225, Thermo Scientific) according to the manufacturer’s protocol, using bovine serum albumin as standards. Protein samples were resolved on 10% SDS-PAGE gels and transferred onto PVDF membranes (IPVH00010, Millipore). Membranes were blocked with 2% BSA at least 1 h at room temperature and probed overnight at 4 °C (1:1000) for the following primary antibodies to PD-L1 (E1L3N), STAT1 (9172), pSTAT1 Tyr-701 (D4A7), SOCS3 (2923S), IDO1 (D5J4E) all from Cell Signaling Technology, or ATP1A1 (ab7671 464.6), from Abcam, and for 1 h at room temperature for GAPDH (6C5), from Abcam. Membranes were further incubated with horseradish peroxidase (HRP)-conjugated secondary antibodies (P044701-2 or P044801-2, Dako Agilent) and visualized with ECL (10754557, GE Healthcare Amersham) on a ChemiDoc MP imaging system (BioRad) or on film (28906836, GE Healthcare) and developed (Xograph). After probing for pSTAT1(Y701), the blot was stripped with Restore PLUS Western Blot Stripping Buffer (46428, Thermo Fisher Scientific) for 15 min at room temperature, blocked with 2% BSA at least 1 h at room temperature, then probed for total STAT1. Films were scanned (HP Scanjet 200), and the following parameters were adjusted for all blots: highlights 35, shadows -16, midtones 0, gamma 1.8. Band intensity was quantified using Fiji 2.0.0-rc-69/1.52p (NIH, Bethesda, MD) (96).

### Intracellular sodium assay

MDA-MB-231 cells were seeded in a 96-well plate at 50,000 cells per well. Cells were treated with 1 U/ml IFN-γ, 6.25 ng/ml TNF ±100 nM ouabain or 150 nM digoxin, 0.05% DMSO. Twenty-two hours after cytokines were added, cells were washed with FCS-free DMEM ±100 nM ouabain or 150 nM digoxin, 0.05% DMSO. Calibration wells and blanks were maintained in fully supplemented DMEM. This was followed by a 2 h incubation with 10 μM SBFI-AM, 0.1% pluronic F-127 in corresponding treatment solutions made up in unsupplemented DMEM. After incubation, samples and blanks were washed twice and topped up with physiological saline solution (PSS – 144 mM Na^+^). Calibration wells were washed with saline solutions of different sodium concentration (0, 25, 50, 75, 100 mM), 20 μM gramicidin was added to the calibration wells only. Fluorescence was read at 340 and 380 nm using a Clariostar (BMG Labtech) plate reader. Fluorescence was measured every 5 min for 25 to 30 min, to observe the timepoint at which the activity of gramicidin was optimal. Data were analyzed by normalizing to blank values and calculating the ratio of fluorescence at 340 nm/380 nm. As quality control, data at 380 nm with a signal-to-noise ratio below 20 were excluded. The optimal timepoint calibration data were plotted in GraphPad Prism v8.3.0 and analyzed using a nonlinear regression (Padé (1,1) approximant) curve. The associated equation was used to determine intracellular sodium concentrations in each sample.

### Statistical analysis

Statistical analysis was performed in GraphPad Prism v9.0.0. Data are represented as mean ± standard deviation, indicating individual replicates where appropriate. Data were analyzed by ANOVA with Tukey’s post-hoc test to account for multiple comparisons, where appropriate. Schematics were made with BioRender.com.

## Data availability

Primary data are available upon request.

## Supporting information

This article contains supporting information.

## Conflict of interest

The authors declare that no conflicts of interest exist with the contents of this article.
