# Supplementary material for: Inhibition of the Na+/K+-ATPase by cardiac glycosides suppresses expression of the IDO1 immune checkpoint in cancer cells by reducing STAT1 activation
Source: J Biol Chem. 2022 Feb 9;298(3):101707. doi: 10.1016/j.jbc.2022.101707 (PMC8902613; doi:10.1016/j.jbc.2022.101707)
Supplement: Supplemental Table S1 [file mmc1.docx]

**Supporting Table 1:** Compounds used for the ion-modulating drug screen.

| **Drug/toxin** | **Target(s)** | **Effect** | **Final concentration (µM)** | **Final DMSO concentration (%)** | **Supplier/Catalogue Number** |
| --- | --- | --- | --- | --- | --- |
| 1- Ethylbenzimidazolinone (1-EBIO) | K_Ca_3.1, K_Ca_2 channels | Activator | 50 | 0.05 | Sigma Aldrich SML0034 |
| 4-aminopyridine | K_v_ K^+^ channels | Inhibitor | 100 | 0.05 | Fluorochem 047207 |
| Amiloride hydrochloride dihydrate | ASIC channel | Inhibitor | 50 | 1 | Alfa Aesar J62168.03 |
| Benzamil hydrochloride hydrate | Na^+^/Ca^2+^ exchanger, ENaC, TRPP3, TRPA1 channels | Inhibitor | 10 | 0.05 | Sigma Aldrich B2417 |
| Bupivacaine hydrochloride | Na_v_, K2P channels | Inhibitor | 100 | 0.05 | Fluorochem M06305 |
| Capsaicin | TRPV1 channels | Inhibitor | 10 | 0.05 | Sigma Aldrich M2028 |
| Carbenoxolone disodium salt | Pannexin-1 (Gap junction channel) | Inhibitor | 3 | 1 | Alfa Aesar J63714.03 |
| Cariporide | NHE1 | Inhibitor | 0.05 | 0.05 | Sigma Aldrich SML1360 |
| Carvedilol | K_v_1.5, K_ir_2.3 K^+^ channels, adrenoceptors | Inhibitor | 5 | 0.05 | Sigma Aldrich PHR1265 |
| Celecoxib | L-type Ca^2+^ and K_v_7 channels | both | 10 | 1 | Sigma Aldrich PHR1683 |
| Charybdotoxin | K_Ca_1.1, K_v_1.2, K_v_1.3 K^+^ channels | Inhibitor | 0.1 | 0.05 | Sigma Aldrich C7802 |
| Diclofenac sodium | K_v_7 channels | Activator | 100 | 0.05 | Calbiochem 287840 |
| Eslicarbazepine acetate | VGSC | Inhibitor | 100 | 0.05 | Spectrum Chemical TCI-E1046 |
| Flunarizine dihydrochloride | L-type and T-type Ca^2+^ channels | Inhibitor | 10 | 0.05 | Sigma Aldrich F8257 |
| Fluoxetine hydrochloride | K_v_4.3 channels | Inhibitor | 10 | 0.05 | Sigma Aldrich PHR1394 |
| Gabapentin | VGCC/ɑ-2-δ | Inhibitor | 2 | 0.05 | Generon A8436 |
| Glibenclamide | TRPA1, KATP channels | Inhibitor | 10 | 0.05 | ApexBio Technology B1296 |
| Iberiotoxin | K_Ca_1.1 K^+^ channels | Inhibitor | 0.1 | 0.05 | Alomone Labs STI400 |
| Icilin | TRPM8 channel | Activator | 1 | 1 | Generon A13560 |
| Loperamide hydrochloride | L-type, T-type Ca^2+^, HCN channels, NMDA, μ-opioid receptors | Inhibitor | 1 | 0.05 | Merck 5081620001 |
| Margatoxin | K_v_1.3, K_v_1.6 | Inhibitor | 0.0005 | 0.05 | Alomone Labs STM325 |
| Memantine hydrochloride | NMDA receptor | Inhibitor | 1 | 0.05 | Alfa Aesar J63830MF |
| Nifedipine | L-type Ca^2+^ channels | Inhibitor | 10 | 1 | Sigma Aldrich PHR1290 |
| NS-1619 | K_Ca_1.1 K^+^ channels | Activator | 1 | 0.05 | Alomone Labs N105 |
| Ouabain octahydrate | Na^+^/K^+^ ATPase | Inhibitor | 50 | 1 | Sigma Aldrich O3125 |
| Phenytoin sodium salt | VGSC, HERG | Inhibitor | 50 | 0.05 | Sigma Aldrich D4505 |
| Ranolazine | VGSC | Inhibitor | 10 | 0.05 | ApexBio Technology A8510 |
| Tetraethylammonium chloride monohydrate | K^+^ channels | Inhibitor | 30 | 0.05 | Sigma Aldrich 86605 |
| Tetrodotoxin citrate | VGSC | Inhibitor | 10 | 0.05 | Alomone Labs T550 |
| TRAM-34 | K_Ca_3.1 K^+^ channels | Inhibitor | 0.5 | 0.05 | Tocris 2946 |
| Veratridine | VGSC | Activator | 10 | 1 | Tocris 2918 |
